# Strengthening breast cancer services in Vietnam: a mixed-methods study

**DOI:** 10.1186/s41256-019-0093-3

**Published:** 2019-01-30

**Authors:** Chris Jenkins, Tran Thu Ngan, Nguyen Bao Ngoc, Tran Bich Phuong, Lynne Lohfeld, Michael Donnelly, Hoang Van Minh, Liam Murray

**Affiliations:** 1 Centre for Public Health, Queen’s University Belfast, Royal Victoria Hospital Site, Institute of Clinical Sciences Block B, Grosvenor Road, Belfast, BT12 6BJ UK; 2 Centre for Population Sciences, Hanoi University of Public Health, Hanoi, Vietnam

**Keywords:** Breast Cancer, Vietnam, Health systems, Cancer, NCDs

## Abstract

**Background:**

Incidence of breast cancer has increased in Vietnam over the past two decades, but little data exists to inform policy and planning. This study examined the organisation and delivery of breast cancer services in Vietnam in order to address the lack of data on detection, diagnosis and treatment.

**Methods:**

We gathered quantitative and qualitative data using an adapted survey-based Service Availability and Readiness Assessment (SARA) tool and semi-structured interviews from healthcare providers in 69 healthcare facilities about the experience and challenges of delivering breast cancer services. We conducted our study across four levels of the health system in three provinces in Vietnam.

**Results:**

The analysis of our data show that a number of areas require strengthening particularly in relation to service availability and service readiness. Firstly, healthcare providers across all levels of the health system reported that service provision was constrained by a lack of resources both in relation to health infrastructure and training for healthcare providers. Secondly, access to timely diagnosis and treatment is limited due to services only being available at the top two levels of the health system. Women living outside the immediate vicinity of such facilities tend to find access more costly and time-consuming, and there is a need to investigate the social, economic, geographic and cultural barriers that may prevent women from accessing services.

**Conclusions:**

Our study suggests that there is a need to strengthen lower levels of the Vietnamese health system in relation to the detection of breast cancer. Provision of some services such as clinical breast examination, advice on self-examination, and conducting ultrasound tests (supported with appropriate training and capacity-building of healthcare providers) at commune and district levels of the health system may reduce the overcrowding and service-delivery burden experienced in provincial and national-level hospitals. Empowering lower levels of the health system to conduct breast cancer screening, which is currently undertaken on an ad hoc basis through higher-level facilities, is likely to improve access to services for women.

## Background

The incidence of detected breast cancer has increased steadily in Vietnam over the past two decades. Data from 2012 indicates an increase from an age-standardised rate of 16.2 per 100,000 in 2002, to 23.0 per 100,000 in 2012 [1], although the extent to which this represents a true increase, or is merely reflective of improved measurement and detection, is unclear. Studies indicate that breast cancer in Vietnam tends to be diagnosed at an advanced stage [1–4], and while qualitative data is limited, this is likely due to poor levels of awareness concerning breast cancer symptoms and difficulties in accessing detection services [5]. Empirical studies are sparse regarding the type, range and location of breast cancer services and how they are organised and delivered across different levels of the Vietnamese health system. There is also little known about challenges that staff face when delivering these services. Therefore, the present study was undertaken to address this lack of information particularly regarding services for the detection, diagnosis and treatment of breast cancer.

**Fig. 1:**
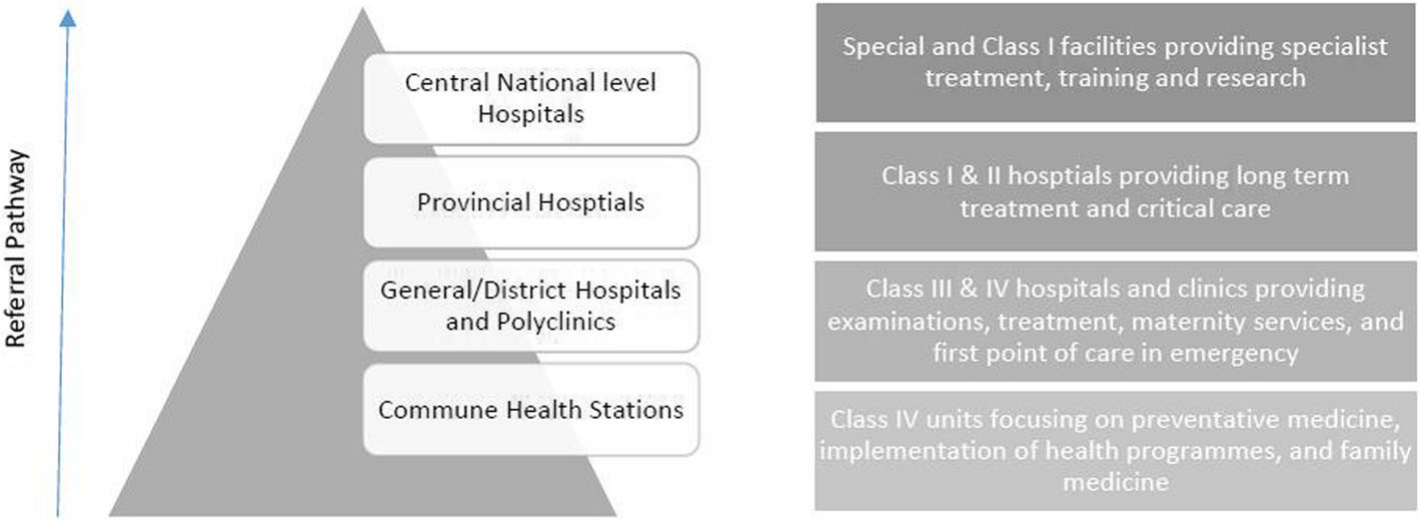
Overview of the structure, class categorisation and functions of different levels of the Vietnamese health system [6]. Adapted from Shillabeer [7]

The Vietnamese health system is organized as a four-tiered healthcare structure, shown in Fig. 1. This structure corresponds with a dual system of categorisation, in which each facility is categorised into a specific ‘class’, based on a score the facility receives on factors such as infrastructure and equipment, human resources, services offered, size, and location.

Facilities are administered by either the Ministry of Health (for facilities at the Central and Special levels), or by regional departments of health. As with most health systems, specialised services are provided at central, national and provincial levels, and often only in urban settings. Commune health stations and general district hospitals are entry points into the health system and provide examinations, referrals and in some cases basic treatment.

This study provides an initial insight into the delivery of breast cancer services in Vietnam. It compliments work initiated by the Ministry of Health to provide a policy framework and guidelines for service delivery for non-communicable diseases (NCDs) in general, and breast cancer specifically [8–10], and supports the Ministry in their continued efforts to broaden and strengthen cancer services.

This study forms part of a wider research programme that aims to make evidence-based recommendations for strengthening breast cancer services in Vietnam. Other studies within this collaboration include a scoping review of relevant studies about breast cancer services in Vietnam [11] and a study of the experience of women using breast cancer services.

## Materials and methods

The study used a concurrent nested (embedded) mixed-methods approach [12] and collected data through a self-administered survey (*n* = 69) and in-depth interviews (*n* = 23) with healthcare providers who worked at facilities across all four levels of the Vietnamese health system (national, provincial, district, commune). Our study gathered data from three provinces, representing the northern, central, and southern regions of the country.

The survey was modelled on the Service Availability and Readiness Assessment (SARA) tool developed by the World Health Organisation [13] and on previous modifications of the tool used in studies in Vietnam [14]. Our modified tool used the SARA template and adapted it to examine services related to a single disease (breast cancer). SARA aims to provide data for policy makers to make informed, evidence-based decisions about the planning and delivery of health services and is used to gather information about service availability, readiness, and service-specific readiness. Service availability is defined as ‘the physical presence of the delivery of services and encompasses health infrastructure, core health personnel and aspects of service utilization’. Service readiness is ‘the availability of components required to provide services, such as basic amenities, basic equipment, standard precautions for infection prevention, diagnostic capacity and essential medicines’. Service-specific readiness is ‘the ability of health facilities to offer a specific service, and the capacity to provide that service measured through consideration of tracer items that include trained staff, guidelines, equipment, diagnostic capacity, and medicines and commodities’.

Following guidelines on designing surveys [15] we conducted an initial round of key informant interviews and carried out a systematic review of available literature on breast cancer services in Vietnam [11]. We generated the items for each of the three SARA-based domains for the modified version of the survey, and then organised the items in the survey to reflect the six building blocks of health systems developed by the WHO [16]. These are: leadership/governance; health care financing; health workforce; medical products and technology; information and research; and service delivery. We also developed an interview guide for use with a subset of healthcare providers who completed the SARA survey.

We pilot tested the survey and interview guide in Bac Giang and Dong Thap provinces, interviewing representatives from the provincial hospital in each province; representatives from two district hospitals; and representatives from two commune health stations in each province. The purpose was to receive feedback on the wording of questions, ordering, format, the content of the surveys, and to use the information provided on breast cancer services in each facility to inform the type of follow up questions we would ask during the interview stage of the study.

### Sample size and sampling techniques

Three relatively homogenous provinces (in terms of household income, ethnic group and population size) in Vietnam (Bac Giang, Hue, & Dong Thap) that are representative of the northern, central and southern regions (Table 1) were included in the study. In addition, facilities in Hanoi were surveyed in order to capture a profile of breast cancer services in a metropolitan urban setting and to facilitate a comparison with service profiles in less densely populated settings.

**Table 1:** Demographic information on sample provinces [17, 18]

	Bac Giang	Hue	Dong Thap
Area (square miles)	382,739.93	506,527.92	337,637.03
Population	1,554,131	1,087,420	1,666,467
Population: Urban/Rural Ratio	145,745/1,408,386 (1:10)	391,112/696,308 (1:2)	295,959/1,370,508 (1:5)
Monthly income (unit: 1000 VND) 1USD = 22,700 VND (December 2017)	Total: 1103.2	Total: 1058.3	Total: 1138.0
	5 quintiles	5 quintiles	5 quintiles
	402.9	329.7	333.7
	629.8	638.3	635.1
	905.7	876.1	871.7
	1247.1	1220.2	1199.0
	2340.0	2235.6	2657.4

Multi-stage cluster sampling [19] was performed to select health facilities at national, provincial, district, and commune level. In each province, staff at the provincial hospital were surveyed and two districts were selected randomly. In turn, the district hospital and 10 commune health stations within each district were selected via random sampling. All communes were selected in districts that had fewer than 10 communes. Table 2. presents the number of facilities at each level in which staff were surveyed. In total, Bac Giang has 9 districts; Hue has 8 districts; and Dong Thap has 11 districts.

**Table 2:** Sampling Plan: Number of health facilities at each health system level

	Number of health facilities at each health system level where staff were surveyed using the modified SARA form				
	Hanoi	Bac Giang	Hue	Dong Thap	Total
National Hospital	1	0	1	0	2
Provincial Hospital	0	1	1	1	3
District Hospital	0	2	2	2	6
Commune Health Station	0	20	19	19	58
TOTAL	1	23	23	22	69

Interviews were conducted with a subset of staff at each of the national, provincial and district facilities that received a survey. In addition, two commune health stations per district were chosen randomly for an interview. The purpose of the interviews was to broaden and deepen the data requested in the survey, verifying information and accuracy, and providing a quality check [12]. Examples of questions included: what do you think are the biggest challenges or barriers facing women when seeking breast cancer services; what challenges do you and other staff face in trying to meet the needs of women with possible breast cancer or a confirmed diagnosis; and what training has the staff at this facility received in the last 12 months on breast cancer diagnosis, treatment, referral etc. (who provided this training, was it useful, and how could it be improved?). A total of 23 interviews were conducted (Table 3.), each lasting between 45 and 90 min.

**Table 3:** Sampling Plan: Number of health facilities where staff were interviewed

	Number of health facilities that participated in research interviews				
	Hanoi	Bac Giang	Hue	Dong Thap	Total
National Hospital	1	0	1	0	2
Provincial Hospital	0	1	1	1	3
District Hospital	0	2	2	2	6
Commune Health Station	0	4	4	4	12
TOTAL	1	7	8	7	23

### Participants

Senior healthcare providers in managerial positions (director of facility, vice-director, or head of oncology department) completed the survey at each facility and participated in interviews at a subset of facilities. Our response rate for both our survey and interviews was 100% within our targeted sample. Due to the need to protect anonymity we did not collect further demographic information on participants in our study, however 14 of the 23 healthcare providers taking part in our interviews reported the length of time they had been working in healthcare. (Mean 17 years. Range 3–32 years).

### Data collection and analysis

A Standard Operating Procedure was designed for all data collection, storage and analysis. Surveys were sent to all selected health facilities and after one week a member of the research team physically collected the survey. If the survey had not been completed when the member of the research team came to collect it, the member of the research team would support the designated member of the health facility to complete the survey. Support focused on how to follow and understand instructions in survey. Specific training was given to each research team member on how to appropriately provide advice and support on completing the survey, and how to avoid leading respondents in how they answered the questions.

In facilities randomly selected to take part in interviews, the research team would also conduct interviews with senior healthcare providers during the same visit. Two female Vietnamese researchers conducted the interviews. All interviews were audio-recorded with participants’ prior consent.

Data from the surveys were inputted into SPSS for statistical analysis. Qualitative data was thematically analysed by the research team. Transcripts were translated, back-translated, and codes to analyse the qualitative data were identified. The data was discussed, categorised and collaboratively analysed by members of the research team [20].

## Results

The results of our study are presented with specific attention to how they corresponded to the areas of service availability and service readiness, with reference to the WHO’s six building blocks for health systems [16]. Following an overview of responses to our survey, we will present a thematic analysis and discussion of the main sub-themes emerging from both our survey results and our qualitative interviews.

Responses to the survey provided by staff working at the district and commune level facilities are presented in Table 4. Given the small number of participants within our study working in national and provincial level facilities we only present their responses within the thematic discussion and review of qualitative data.

**Table 4:** Overview of results from survey

		Facility Type	
Service	Provided	District (*n* = 6)	Commune (*n* = 58)
Health Promotion (awareness raising)	Yes	5 (83.3%)	52 (89.7%)
	No	1 (16.7%)	6 (10.3%)
Training to self-examine	Yes	4 (66.7%)	42 (72.4%)
	No	2 (33.3%)	16 (27.6%)
Clinical Breast Examination	Yes	6 (100%)	34 (58.6%)
	No	0 (0%)	24 (41.4%)
Opportunistic Screening	All the time	1 (16.7%)	8 (13.8%)
	Sometimes	1 (16.7%)	25 (43.1%)
	Rarely	1 (16.7%)	7 (12.1%)
	Never	3 (50%)	13 (22.4)
	Missing	0 (0%)	5 (8.6%)
Ultrasound	Yes	5 (83.3%)	3 (5.2%)
	No	1 (16.7%)	55 (94.8%)
Diagnosis (pathological confirmation)	Yes	0 (0%)	0 (0%)
	No	6 (100%)	58 (100%)
Treatment	Yes	0 (0%)	0 (0%)
	No	6 (100%)	58 (100%)
Palliative Services (inc. use of morphine)	Yes	4 (66.7%)	13 (22.4%)
	No	2 (33.3%)	45 (77.6%)
Resources/Training			
Do you have the staff, resources, and equipment to provide all breast cancer services you are authorised to provide?	Yes	3 (50%)	33 (56.9%)
	No	3 (50%)	22 (37.9%)
	Missing	0 (0%)	3 (5.2%)
Cancer and breast cancer training provided to staff at the facility	Yes – General Cancer Training	3 (50%)	24 (41.4%)
	Yes – Breast Cancer specific training	1 (16.7%)	10 (17.2%)
	No	2 (33.3%)	24 (41.4%)
Community Screening			
Supports community-based screening activities	Yes	3 (50%)	44 (75.9%)
	No	3 (50%)	11 (19.0%)
	Missing	0 (0%)	3 (5.2%)
*If YES, what roles are undertaken? (percentage as total percent / valid percent)*			
Lead the operation	Yes	2 (33.3% / 66.7%)	13 (22.4% / 29.5%)
	No	1 (16.7% / 33.3%)	31 (53.4 / 70.5%)
	Missing	0 (0%)	14 (24.1%)
Technical support (supporting the actual delivery of screening services, lead by a higher level health facility)	Yes	2 (33.3% / 66.7%)	4 (6.9% / 9.1%)
	No	1 (16.7% / 33.3%)	40 (69.0% / 90.0%)
	Missing	0 (0%)	14 (24.1%)
Administrative support (advertising screening, patient registration etc.)	Yes	0 (0%)	32 (55.2% / 72.7%)
	No	3 (50% / 100%)	12 (20.7% / 27.3%)
	Missing	0 (0%)	14 (24.1%)

### Service availability

In relation to breast cancer, and illustrated within our table, the availability of services at lower levels of the system is limited and variable. Specialised services are only available (and only authorised by the Ministry of Health) at the higher-level facilities in the Vietnamese health system (national and provincial levels).

None of the surveyed district hospitals or commune health stations offer breast cancer diagnostic or treatment services, however one participant in our interviews described that their district health facility could provide simple surgical procedures (such as lumpectomy). While all district facilities reported the ability to conduct clinical breast examination (CBE) and five reported conducting ultrasound tests (83.33%), in reality most participants within our interviews described breast cancer services and care at district and commune levels of the health system as minimal. CBE is often only provided at district facilities when requested and to those with a family history.

At the higher levels of the health system, all three provincial hospitals surveyed reported diagnostic capacity, however only two of them could perform surgical interventions (mastectomy and breast conserving surgery) and chemotherapy. Radiotherapy services were only provided in one of the three provincial hospitals surveyed. Overcrowding and a lack of beds were reported at all upper levels of the health system and a number of respondents at higher levels of the health system also reported a lack of human resources capacity, with either insufficient numbers of staff, or insufficient training and knowledge of staff concerning breast cancer. One doctor at a provincial health facility summed up the challenges, stating:

*In short, it’s only me. I do my best to do screening and surgery. And once a week I will be available at the clinic on Tuesday mornings to examine breast cancer patients and to do screening. When I diagnose someone having cancer, the nurses are shocked and sad, as if the patients will die for sure. They do not understand. I am sad that even staff of a provincial hospital think like that.*

The shortage of trained staff was communicated by respondents across the health system. At commune level there is often only one doctor per facility, meaning if that doctor is absent, ill, or on leave, then services will be reduced. At higher levels shortages put pressure on staff to deliver services effectively and restrict the amount of time that can be spent interacting with patients. A respondent from a district facility highlighted this problem in relation to providing self-examination training to women, stating, ‘*Sometimes there are too many patients. There is about 100 patients per day. If there is time we will instruct them, otherwise we wouldn’t.’*

Given the potential sensitivity of breast cancer, and the invasiveness of procedures to detect, diagnose and treat, there may also be challenges concerning male doctors conducting these procedures. It was described that often a women’s preference would be for a female doctor to conduct these procedures and examinations, and yet at some facilities there were insufficient numbers of trained female staff. One district level facility reported just one female doctor with the training to conduct CBE.

The provision and availability of screening services appears to vary widely across our sample. In some areas consistent screening activities appear to be implemented with a wide range of health care facilities involved in their delivery. Both national level facilities surveyed reported conducting and/or supporting community-based screening activities. One facility reported leading the operation, while the other reported providing administrative and technical support. One respondent from a national level facility reported that staff from the hospital would directly provide screening services (CBE only) at commune level facilities (10 per year) while also training staff at lower levels of the system to conduct examinations independently. The respondent also stated that many screening services were offered directly at the hospitals, with different organisations invited to bring their staff and members for screening.

At provincial level, two of the three surveyed facilities reported active involvement in community screening programmes, and both reported to be leading the operations. District hospitals in one province described organising three screenings per year, with an additional screening organised by a higher level facility every year (funding dependent). In another province, the provincial level facility reported supporting community-based screening four or five times every year. The senior healthcare provider from the provincial hospital reporting no involvement in screening stated, *‘We’ve never organised a programme coming down to the communities’*, and that they were also unaware of any other organisations conducting screening programmes in the province.

This trend of facilities from higher levels of the health system leading in the implementation of screening programmes is commonly reported, with the role of the lower levels of the system to support in organisational, administrative and technical aspects of screening. This includes recruitment, advertising, and providing the venue for screening to be conducted. In total, at commune level, 75.9% of respondents stated that they had been involved in community-based screening activities. Only 6.9% reported having a technical role in the screening process (for example, supporting examinations). A number of communes (24.1%) stated there were no screening programmes for breast cancer at all in their area.

### Service readiness and service-specific readiness

While services may be available at different levels of the Vietnamese health system, resource constraints both in terms of equipment and a lack of capacity of staff to detect, diagnose, and treat breast cancer were frequently reported across all levels of the health system. Shortages in staff availability is often compounded by a lack of service-specific trained staff. In terms of equipment and physical resources, broken or old ultrasound machines, or ultrasound machines without the capacity to screen the breast, were reported at both commune and district levels. At higher levels of the health system a shortage of equipment was described particularly in relation to radiotherapy capacity. A lack of educational materials was reported particularly at commune level, which restricted the ability of staff to implement health promotional activities.

Analysing the ability of the commune level to provide screening and detection services we found that only 58% of respondents (*n* = 58) said that they offered clinical breast examination to women attending the clinic. For opportunistic screening, 14% reported that they ‘always’ conducted opportunistic examinations; 43% stated ‘sometimes’; 12% ‘rarely’; 22% ‘never’; and 2% ‘don’t know’. Key informants and study participants involved in our data verification process, however, felt that reporting on the frequency of both clinical examination and opportunistic screening may be exaggerated. Respondents in every interview stated that opportunistic screening does not occur, and there was general consensus within the research team that the term ‘opportunistic screening’ was poorly understood throughout the interviewing process.

A lack of visible women with breast cancer was a theme reported at commune level, and while most of the commune level staff surveyed reported providing self-examination training (72.4%), the commune health stations role within the health system in regards to breast cancer appears to be limited to occasional CBE on the request of the patient; health promotional activities; and supporting the higher levels of the health system in administrative and logistical tasks if a screening outreach programme is implemented in the area.

Consistently respondents from commune health stations stated that women with breast cancer do not present at their facilities. *‘We don’t see patients. We were trained by the Ministry of Health (to conduct CBE) but there were no patients who came for examination’.* Another respondent stated, *‘It takes a very long time for someone to come for an examination. Sometimes it can be two months without doing one’.* One respondent stated that in the six years they had been working at the commune level there had never been a breast cancer patient in the area. This illustrates the level of disconnection between the commune level and women with breast cancer. Our data suggests that the commune level of the health system is regularly bypassed, either due to its lack of capacity to provide services, or because women are aware that the services they require are located at the higher levels of the health system.

A lack of training in regards to both breast cancer broadly (at commune level) and more specifically on how to diagnose and treat breast cancer (district and provincial levels), were evident themes in both our survey and interviews. At commune level there are particular challenges related to levels of knowledge and awareness of staff about breast cancer, including symptoms and treatment options. One respondent commented on the lack of training for staff at the facility, stating: “*Years ago we did have training on breast cancer. But it was a very long time ago. I can’t remember when it was. Recently, there has been no training about breast cancer”.* Another respondent said, *“Our capacity and knowledge is limited. How can we provide others with the correct information?”*

Another respondent from a commune health station noted that while it was unlikely that staff would be expected to provide specialised knowledge, it was still important to have a general understanding about breast cancer symptoms and the diagnostic and treatment pathway. Given that a women’s first interaction with the health system may be at commune level, staff need to recognise breast cancer symptoms and respond appropriately. The respondent stated:

*Being the jack-of-all-trades, we are not specialised in anything, but it’s important to update our information regularly to always know of the basics to be able to consult the patient in the best possible way.*

In relation to screening activities, challenges were reported related to transporting the necessary equipment for screening, meaning that most screening activities were only CBE. Therefore, while screening may be available, it is not being supported with appropriate or sufficient physical infrastructure. By conducting screening within commune health stations problems were also reported relating to having enough private rooms for examinations.

### Geographic variations

As previously reported, at the provincial level there are variations in the availability of services particularly in relation to surgery, chemotherapy and radiotherapy. No specialised services (surgery, chemotherapy, radiotherapy) were reported in Dong Thap. Surgery and chemotherapy were only reported at two of the three surveyed provincial hospitals, while radiotherapy is only offered at one.

At district level, variations across many services were reported. For example no facilities in Hue reported providing palliative care services, while all facilities in both Bac Giang and Dong Thap reported have provision for palliative care. All facilities in Hue, however, reported having enough staff, resources, and equipment to provide services for breast cancer effectively in comparison to none in Bac Giang, and one of two in Dong Thap. Neither facility in Dong Thap reported involvement community-based screening activities.

Commune health stations in Hue province reported higher services availability and readiness than both Bac Giang and Dong Thap. All nineteen commune health stations in Hue reported supporting community screening activities, while seven communes and four communes in Bac Giang and Dong Thap reported taking part in no community screening activities. Communes in Hue provinces equally reported higher availability of training provided to women on self-examination (84.2%), and having enough staff, resources and equipment to provide effective services (68.4%). All other services were reported equally across the three provinces. This variation between different regions of the health system may be interpreted as indicating that there is a need to give considered attention to the allocation of resources and, generally, to try to ensure that there is a good match between needs for care and service availability.

### Participant views on priorities for service strengthening

Priorities for how to improve breast cancer services followed directly from the challenges reported in the survey and described in the in-depth interviews. In our survey we asked three variants on how respondents would improve breast cancer services. We asked for general recommendations; the ‘easiest to implement’ recommendation; and the ‘most important’ recommendation to improve the delivery and organisation of breast cancer services. All the questions were open (blank box) and respondents were encouraged to give multiple responses.

Increased availability of training on breast cancer was commonly prioritised across all three variants in the survey. A total of 44 (total *n* = 69) respondents mentioned strengthening capacity and training in the general recommendations section, with 17 and 18 respondents respectively recommending human resources strengthening and training in the ‘easiest to implement’ and ‘most important’ variants of the question. ‘Training’ was described in broad terms at commune level, from training on how to conduct a clinical breast exam, to providing training on using ultrasound, to training on communication skills for health promotional activities. At district level and provincial levels the focus was on how to strengthen the facility’s capacity to conduct more specialised procedures. A number of respondents highlighted strengthening service-specific capacity at district hospitals to conduct pathological tests and more complex surgical interventions.

Other commonly reported general recommendations were to extend community-based screening and/or create an annual screening programme (*n* = 29); to improve and increase communication on breast cancer to promote awareness of symptoms (*n* = 25); and to focus on the provision of more specialised equipment (often ultrasound machines) to the lower levels of the health system (*n* = 13). The need to develop community-based screening, either standalone or aligned to pre-existing programmes on cervical screening, was strongly emphasised by a number of respondents. Discussing the need for screening outside of formal healthcare facilities, one clinician stated that, *‘If we have screening in the community women will feel more comfortable. They need someone to listen to their concerns and provide proper counselling for them’*. It was felt that screening programmes need to be better financially supported, and that healthcare providers organising screening events could make better use of International Women’s Day and Vietnamese Women’s Day to promote breast cancer screening activities.

## Discussion

Three interconnected themes were identified within our thematic analysis of the data: a lack of breast cancer service availability and service-specific readiness across all levels of the health system; a lack of capacity and resource constraints to provide breast cancer services; and a lack of systematic and integrated population screening services. These interconnected themes indicate the need for systems strengthening and integration. Comparing our results to the WHO’s building blocks for health systems [16] (governance; financing; workforce; technologies; information and research; and service delivery), we can see that there are opportunities for strengthening across all six areas.

At a governance level, holistic systems planning and piloting of interventions should be further explored in the strengthening of breast cancer services in Vietnam, as should the development of a strategic breast cancer control and treatment plan. The Ministry of Health has outlined within its strategic plans for the control and prevention of NCDs [8] the objective of reorganising systems ‘for prevention, early detection, diagnostics, treatment and management of NCD from central level to commune level nationwide’, and that ‘90% of commune health stations and equivalent health care facilities (should) have enough essential equipment and drugs for prevention, detection, treatment and management of relevant cancers’ [8]. With the proper resourcing and capacity-building it has also been recommended by the Breast Health Global Initiative that many breast cancer services can be provided outside of central high-level facilities with appropriate skills-building training to detect breast cancer being provided to the general healthcare workforce [21]. In Vietnam, while some training has been provided to staff at lower levels of the health system to detect and diagnose breast cancer, there remains a need for systematic capacity-building at commune and district levels to sufficiently support breast cancer service provision.

The current vertical and central organisation of breast cancer services in Vietnam may limit accessibility and effectiveness. Most clearly, the overcrowded nature of oncology units studied at provincial and national levels indicate the need to diversify where women with breast cancer are treated within the system. Extending the roles and responsibilities of district and commune health stations (with the appropriate support for these facilities to provide services), and investing in rural health infrastructure [7], could have a significant impact on improving breast cancer services. As stated by Yiengprugsawan (et.al) in their study on reorienting the delivery of NCD services in Malaysia, Sri Lanka, and Thailand, ‘NCD care requires integration across all levels of health care: primary care screening of risk factors; timely intervention at secondary and sometimes tertiary and rehabilitation levels, and hospital discharge referrals back for management by primary care’ [22].

Authorising and supporting lower levels of the Vietnamese health system to conduct examinations and diagnostic tests may have a positive impact both in terms of reducing in-direct costs and travel for women in rural districts and in reducing overcrowding in central and provincial hospitals. While capacity-building has been supported by the Ministry of Health across the Vietnamese health system, additional work needs to be completed on ensuring services at lower levels (commune, district and provincial) meet the requirements of women with breast cancer in order to encourage women not to bypass them in favour of seeking diagnostic and treatment services at the central and national levels of the health system.

The decentralisation of health systems in low and middle income contexts has been a significant trend in health systems reorganisation for forty years. In their systematic review of literature on decentralisation, Muñoz (et.al) [23] state, ‘Experiences suggest the decentralisation of governance, financing and service-availability, could have positive impact on the system’. While challenges were noted in decentralising resource management and that qualitative data suggests a ‘heterogeneous picture’ concerning impact, the authors argue ‘lessons learned from the decentralisation processes in LMICs suggest that factors such as adequate mix of technical skills at the local level to perform decentralised tasks, effective decentralisation of decision-making to the periphery, and political leadership are key factors for a successful decentralisation process’ [23].

The results of the survey and interviews indicate that there is a need to give particular attention to ways in which to develop cancer services at commune and district levels to increase their visibility, proximity and access. Ideally, detection, screening, and referral services for breast cancer at the lower level of the health system should be provided as a comprehensive and integrated package, with specialised services provided by well-resourced and multidisciplinary teams at higher levels of the health system [24].

It was clear in the priorities provided from healthcare providers that a focus on training and skills development was crucial to increase capacity of the workforce to diagnose and treat breast cancer. This priority has also been recognised in previous publications from the Ministry of Health and the World Health Organisation [9, 25], is highlighted within the WHO’s health systems strengthening frameworks [16], and is also supported by studies that interviewed healthcare providers at commune health stations in Hanoi [26]. This strengthening of human resources should happen systematically and simultaneously with other efforts to strengthen breast cancer services across all six areas of the health system. Our study showed a clear demand from healthcare providers at the lower levels of the system to be given more autonomy, responsibility, and skills to contribute to detection, diagnosis and treatment activities.

A more systematic and strategic approach to screening should be explored. Screening was reported as ad hoc, localised, and infrequent by many participants in our study. Dong Thap province reported an absence of screening activities, and the health inequalities this may create should be addressed. Even the most systematically organised programmes, described by a respondent from a national facility, only reach an estimated 10 communes per year. There may be problems with the funding model for screening. Most screening activities are conducted by national or provincial level hospitals, the costs of which come from their autonomous budgets. This may limit scope. There is no central fund or allocation of money to conduct screening. The quantity of screenings conducted is dependent on how much money individual hospitals are able or willing to contribute towards them. One respondent described screening programmes as ‘charity’ from the hospital. Centralised planning and funding of screening programmes should be further explored, and population-based models for breast cancer screening should be developed [27].

The overall picture is one of disjointed, unintegrated community/population screening, supported in an ad hoc fashion by the upper levels of the health system. There needs to be a more coherent, integrated and systematic approach to screening across the country. As one respondent from a commune station, when asked about the strategy for breast cancer screening, responded, *‘I mean, there’s no plan for breast cancer screening’.*

Furthermore, there is a need for integrated and robust financing structures to support breast cancer services. Economic barriers to accessing services were described as significant by multiple participants within the study, and as previously discussed, there is a lack of central financial planning for screening services. Given the complexity with the insurance system, the Ministry of Health has initiated changes to streamline patients into the central levels of the health system. While this may make for more efficient patient referral pathways, it may also contribute to the overburdening of the top levels of the system. In the past, in order to claim health insurance, patients had to progress through the health system in a linear sequence. Often, patients would bypass the primary levels, going directly to tertiary care. If they needed extensive treatment, they would then return to the commune level and go through formal referral. A new Government directive [28], made it possible for patients to bypass the Commune level and enter at District level. While in some ways this avoids duplication of work, and provides flexibility in an otherwise rigid linear system, it may also further contribute to the underutilisation of primary level health services. This may additionally compound the lack of capacity at primary levels to provide breast cancer detection and referral services.

This study has attempted to provide primary data to help inform decisions on the future of breast cancer service provision. More comprehensive data collection, and monitoring and evaluation systems would be invaluable for breast cancer systems strengthening. As noted in previous studies, Vietnam still lacks a comprehensive and integrated national cancer registry [27], representing a further gap in terms of health system strengthening. Health financing studies, and qualitative studies that explore the experience of women with breast cancer in accessing and using screening and treatment services would be equally useful and important. Systems strengthening for breast cancer should also consider the knock-on impacts of strengthening a specific service, and be conscious of how improvements in one area can either benefit or harm services in other parts of the health system. It is important to remain cognisant that ‘all systems are contained or nested within larger systems’ [29].

## Conclusion

Resource constraints and the concentration of resources, staff and equipment for diagnosing and treating breast at higher levels of the health system, creates significant challenges in effectively detecting, diagnosing and treating breast cancer in Vietnam. Our study suggests that there should be concerted efforts to strengthen commune and district levels of the health system in relation to detection of breast cancer. Specific attention should be given to increasing autonomy and support for commune level health stations to conduct screening activities; providing clinical breast examinations and advice on self-examination; and the extension of regular and targeted breast cancer-specific training for commune and district level health care staff.

### Limitations

Our survey tool, while developed with extensive testing through two pilot studies, was still unable to capture as much information as we had hoped. We received returned surveys with incomplete data on certain sections. Due to the lack of systematic data collection of patient records, many of the questions were answered using estimations by those completing the survey, and therefore only represent broad benchmarks for, eg. frequency of opportunistic screening.

Due to our limited sample size we were unable to test with confidence whether our findings, particularly at the upper levels of the health system are reflective of specific geographic trends or patterns. Our small sample size allowed us to only look at geographic differences between the northern, central, and southern areas in fairly descriptive terms, preventing us from conducting in-depth analysis of any variations.
